# Annotations

**Published:** 1887-03-05

**Authors:** 


### ANNOTATIONS.

Miss Manson and Trained Nurses.

We have reason to believe that Miss Manson's paper, recently read at the Hospitals Association, has given rise to much lively discussion, and to not a little criticism of an adverse character. With the main propositions of the paper most people practically acquainted with the subject will be disposed to agree. That thoroughly trained nurses are indispensable for scientific medical practice; that the general hospitals are the natural training grounds for such nurses; that nurses should be trained to such a degree of perfection, as to merit and receive what is equivalent to a *degree in nursing* from their several hospitals; that this degree or diploma should entitle them to registration; and that each large hospital should have a nursing home in connection with it, from which the public could be supplied with duly accredited nurses at all times—these are propositions with which, as we have said, few will disagree, who have much knowledge of the subject. Miss Manson, however, indulged in some remarks which were not calculated to flatter existing nursing institutions, or to commend them to public confidence. Some of these were spoken of as farming out nurses for their own profit, and of others it was said that they did not exercise all the care they might in the selection of their nurses. An impression was produced by the paper, which was intensified by some of the speakers who took part in the after-discussion, that the present nursing institutions were, on the whole, very unsatisfactory, and that the need for some such changes as Miss Manson suggested was very urgent. That is, perhaps, hardly a fair presentation of the actual facts of the case; and, indeed, when a speaker has a special cause to plead, some licence must be allowed for over-statement. Possibly Miss Manson, in her enthusiastic zeal for needed reforms, did not quite do justice all round. There cannot be the least doubt that there are in London and elsewhere, nursing institutions which are conducted on honourable principles; which select their nurses with all possible care; which treat them kindly and liberally, and which deserve both the confidence and the gratitude of the public.

Drinks Wanted.

Tea, coffee, and cocoa; beer, wine, and spirits. There we stop! If a man does not like tea, or if it makes him dyspeptic, has he not the choice of coffee and cocoa? But coffee is poison to some people, and cocoa at best is only to be taken on sufferance. The truth is that for table use we have only two really palatable beverages, tea and coffee. Yet we have invented the steam-engine, and the spinning-jenny, and the bicycle, and the cock that crows for a penny—with various other ingenious and useful things. Among drinks that may be classed as merely luxurious or medicinal, we are just as badly off. Beer, wine, and spirits, these are the three. Beer is only a luxury under peculiar circumstances. Spirits never ought to be taken except medicinally; and so we are shut up to wine. What poisonous and undrinkable stuff much of it is! Why can we not have something which will give a gentle and ethereal stimulus, without making us frantic and quarrelsome, or stupid and helpless? The teetotallers have invented some fluids which they call "wines"; but of all the abominations which have ever been called by classical names, surely non-alcoholic wines are the most abominable. Will nobody come to the rescue, and raise for himself both a fortune and a monument, by inventing a really pleasant and nutritious drink?

Who Stands Sponsor for the Matrons?

"Quæstor" is anxious to know who "goes bail for hospital matrons"? He suggests that a good deal of what he calls "fuss" is made about the training and registering and certifying and "badging" (he spells it "badgering") of nurses, so that the public may be quite sure that they really have the genuine article; bnt he sees no efforts made at all towards certifying the hospitals and the public that their much trusted matrons are of the right stamp. We fear that Quæstor is inclined to be impertinently inquisitive. A rude young fellow, who had recently disagreed with his superiors at his preliminary scientific examination, sarcastically desired to know who examined the examiners. Quæstor's question is of the same uncivil character. Still, it does certainly open the gate into a wide field of speculation. Matrons are peculiar persons. They live in, and preside over a little world of their own. In their limited sphere they have large powers, and it is sometimes hinted that those powers are not always used with perfect impartiality. It is suggested by Quæstor that all women are more or less governed by feelings, and that many of them have rather spiteful sensibilities. He is of opinion that hospital matrons are no exception to the rule. He asks us to "let in a little light upon the matron question, if we can." We are not aware that any Diogenes lantern is particularly needed at the present time; but if Quæstor or any other person knows of any undoubted facts which warrant his rather cynical surmisings, we shall be glad to be informed of them, and to do what lies in our power in the interests of "justice to all."

---

The Glasgow Dental Hospital is a young and successful institution. Though it is but two years old, it is already able to pay its way and to go forward with a small balance in hand. Last year no less than 6,825 patients were treated. The teeth play a very important part in the economy of our health, and the rapid strides which dentistry has made during the past few years, point to the recognition of this fact. Sound teeth mean not only freedom from pain, caused directly from dental decay, but freedom from many forms of disease besides.

